# A prototype smartwatch for monitoring dynamic, compound and plyometric exercises in cancer prehabilitation: a development and validation study

**DOI:** 10.3389/fdgth.2026.1846350

**Published:** 2026-06-03

**Authors:** San San Tay, Hanushyah Devi, Shannon Yap, Abbas Bin Zainul Abideen, Xuan Han Koh, Daniel Leong

**Affiliations:** 1 SingHealth-Changi General Hospital, Singapore, Singapore; 2 SingHealth-Eastern General Hospital, Singapore, Singapore; 3 Duke-NUS Medical School, Singapore, Singapore

**Keywords:** cancer prehabilitation, compliance tracking, exercise monitoring, mHealth, plyometric exercise, smartwatch, wearable technology

## Abstract

**Background:**

Cancer prehabilitation programmes increasingly rely on home-based exercise interventions, yet objective monitoring of exercise compliance remains challenging. Current commercial wearables focus primarily on aerobic activities and lack capability for monitoring dynamic, compound, and plyometric exercises essential for cancer prehabilitation.

**Objective:**

To develop and validate a prototype smartwatch capable of monitoring specific prehabilitation exercises with objective compliance tracking.

**Methods:**

We developed a prototype using the M5StickC PLUS2 development board with integrated 6-axis inertial measurement unit. Seven standardised cancer prehabilitation exercises were monitored using a novel Rep Error Height (REH) scoring algorithm. The study comprised two phases: algorithm development with 6 healthcare workers providing reference waveforms, and validation with 20 healthy volunteers performing exercises under observation and at home for 4 days. Usability was assessed using the adapted mHealth App Usability Questionnaire (MAUQ).

**Results:**

Algorithm performance demonstrated high correlation (88.9%–98.8%) between visual and algorithmic repetition counting across all exercises. Median compliance rates exceeded 90% overall, though specific exercises showed lower compliance on certain days. Usability assessment revealed mean overall scores exceeding 5.0, with ease of use rated most favourably. However, only 35.7% of participants rated device usefulness above 5.0, primarily due to lack of real-time feedback mechanisms.

**Conclusions:**

The prototype has the potential to address the gaps in current wearable technology for cancer prehabilitation monitoring. Whilst demonstrating high accuracy for exercise detection, algorithm refinement is needed to accommodate diverse movement patterns and variations in exercise execution.

## 1 Introduction

Cancer prehabilitation encompasses the period between diagnosis and initiation of acute treatment, aiming to reduce treatment-related morbidity, expand therapeutic options, and enhance physical and psychological outcomes (1). The COVID-19 pandemic accelerated the adoption of technology-enabled prehabilitation programmes, aligning with broader healthcare trends towards telemedicine and telehealth applications (2, 3). This shift has generated substantial interest, research and application in telemedicine (4).

Our institution developed a hospital-associated, home-based prehabilitation programme targeting patients with newly diagnosed gastrointestinal and urological cancers awaiting surgery (5). This multimodal intervention incorporated medical optimisation strategies, individualised exercise prescriptions, and nutritional and mental health support. Patient compliance with home exercise programmes was monitored through weekly to fortnightly telephone consultations with the programme coordinator. Initial evaluation demonstrated significant improvements in functional capacity and mental health outcomes.

In November 2021, we integrated a cancer prehabilitation exercise diary into Health Buddy, our regional healthcare mobile application (Singapore Health Services). Whilst a pilot study demonstrated the acceptability, feasibility, and safety of Singapore's first smartphone application for exercise prescription in cancer prehabilitation (6), a critical limitation remained: the inability to objectively monitor exercise compliance.

Remote prehabilitation programmes face inherent challenges with adherence due to lack of direct supervision (7). Exercise intensity and adherence are critical determinants of successful outcomes (8), yet continuous in-person supervision is impractical due to resource constraints. For patients suitable for home-based rehabilitation, incorporating objective compliance monitoring could substantially improve programme effectiveness through adherence tracking, feedback mechanisms, and technique guidance (9).

**Figure 1:**
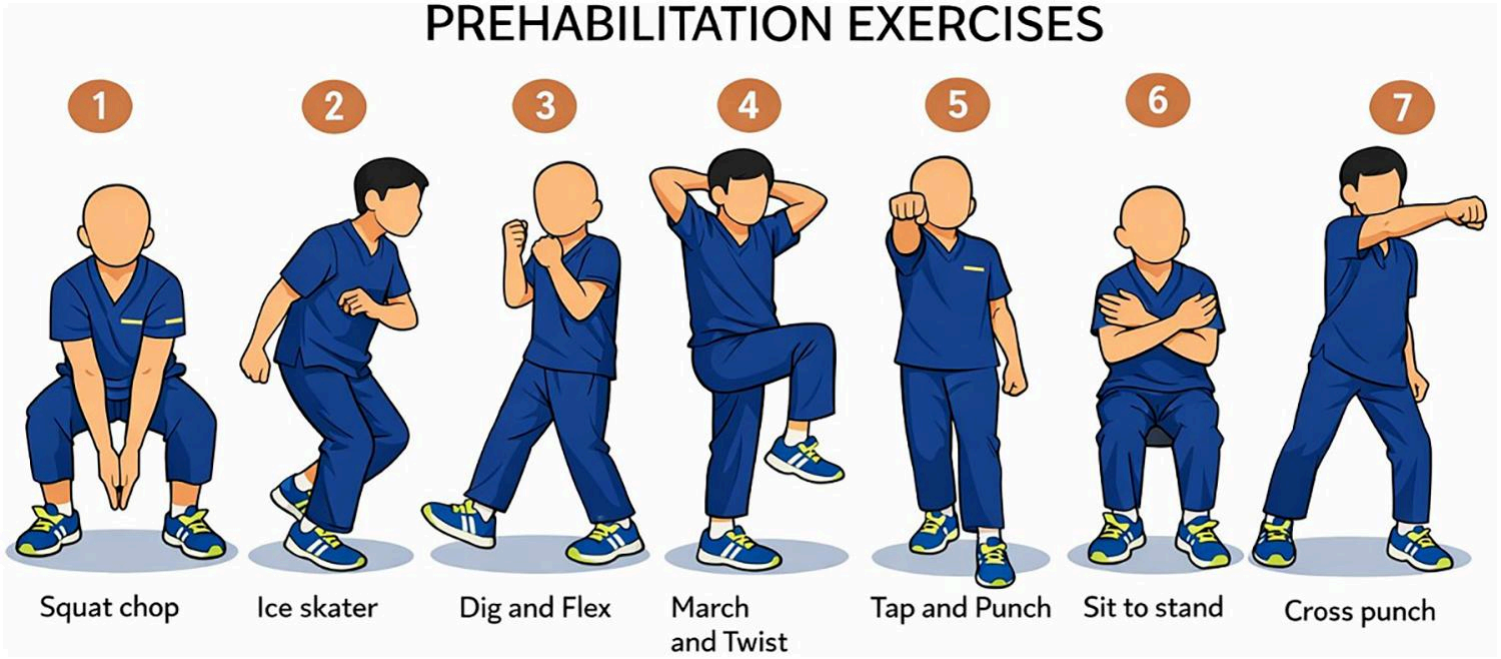
Illustration of the 7 cancer prehabilitation exercises.

The American Cancer Society nutrition and physical activity guideline for cancer survivors recommends both aerobic exercises and resistance training (10). Beyond aerobic activities such as walking, jogging, cycling, and swimming, rehabilitation physicians prescribe dynamic, compound, and plyometric exercises for strength training as these exercises can be performed in the comfort of the home. The Cancer Prehabilitation Exercise Diary comprises seven standardised exercises: squat chop, ice-skater, sit-to-stand, tap and punch, cross punch, march and twist, and dig and flex (Figure 1). These exercises target different physiological adaptations—for instance, ice-skater exercises improve balance whilst squats and sit-to-stand movements enhance lower limb strength. Differentiating training volumes across exercise categories is crucial for programme optimisation. Furthermore, lower limb strength has been correlated with prognosis (11, 12) and balance has been correlated with quality of life in cancer survivors (13).

Contemporary wearable fitness trackers utilising accelerometers and exercise classification algorithms are optimised for aerobic activities but demonstrate poor discrimination between calisthenic and plyometric exercises fundamental to strength training protocols (14). This represents a significant gap across the fitness tracking ecosystem. Commercial Internet-of-Things devices lack programmability and customisation options without proprietary licensing, and additional sensor integration is often impossible.

A recent scoping review revealed that available wearables primarily monitor step counts and distinguish between activity and rest periods (15). Consequently, off-the-shelf devices are inadequate for monitoring dynamic, compound, and plyometric exercises. We aimed to address this limitation by developing a prototype smartwatch capable of asynchronous exercise frequency and accuracy monitoring as proof of concept. The development of the algorithm is described herein.

## 2 Materials and method

### 2.1 Ethics approval and trial registration

This study received approval from the SingHealth Centralised Institutional Review Board (CIRB 2024/4048) and was registered with ClinicalTrials.gov (NCT06970717). All participants provided written informed consent.

### 2.2 Exercise protocol

**Figure 2:**
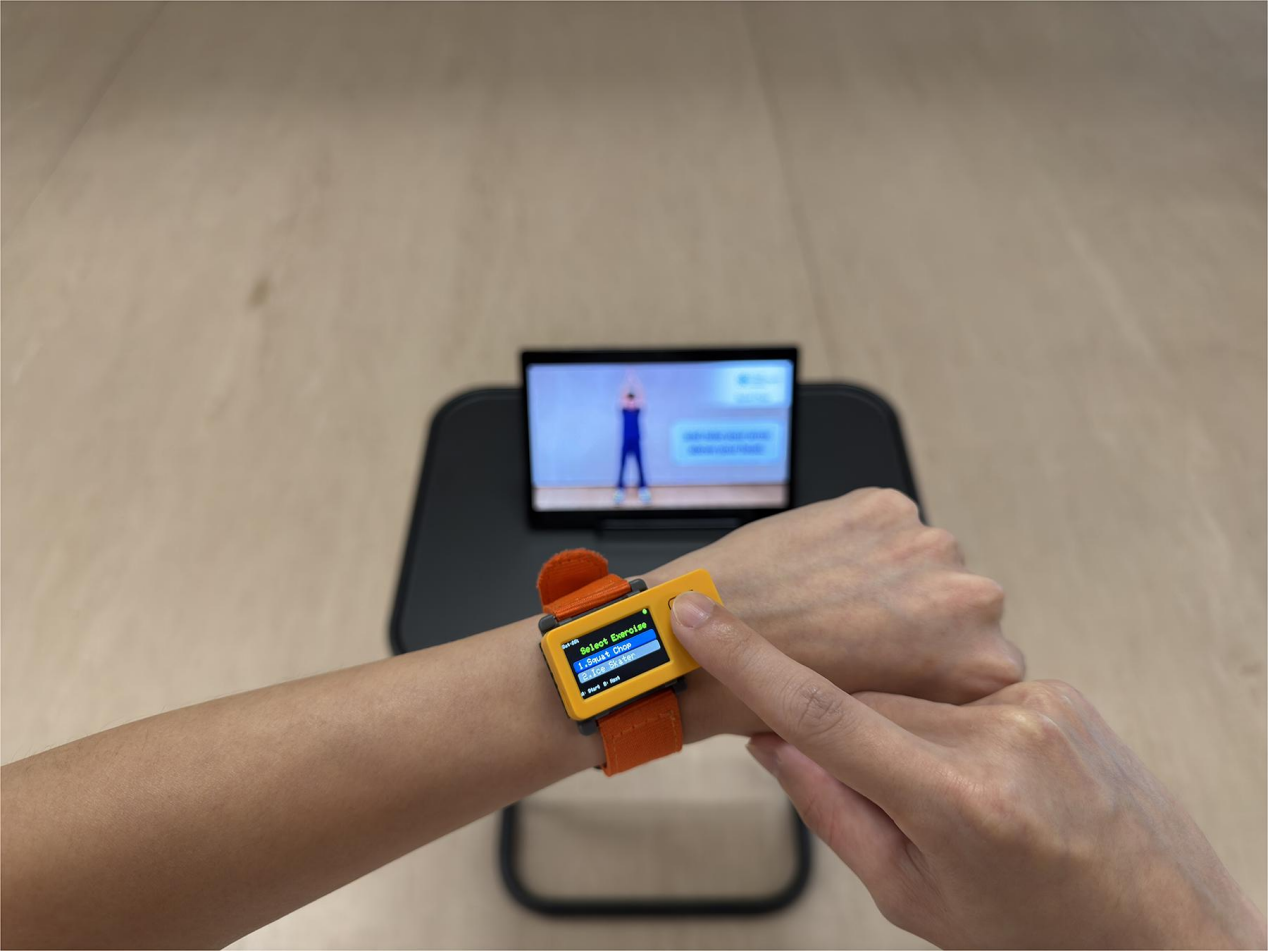
Smart watch in use.

The Cancer Prehabilitation Exercise Diary comprised three repetitions per exercise, which were looped and uploaded to a tablet interface. Participants performed 10 repetitions of each exercise across three sets whilst the smartwatch synchronised with the tablet simultaneously (Figure 2).

### 2.3 Algorithm development

Six healthcare workers provided written consent and participated in performing the seven exercises for waveform collection and simultaneous videography. Healthcare Worker 1 (HCW1), who featured in the original Health Buddy exercise videos, provided the reference waveform, hereafter termed the “golden sample”, for algorithm calibration. The algorithm was developed across four technical stages.

#### 2.3.1 Stage 1: signal acquisition and reference selection

Motion data were acquired using the M5StickC PLUS2 wearable device, which integrates an inertial measurement unit (IMU) specifically the MPU6886 — comprising a 3-axis accelerometer and a 3-axis gyroscope. These sensors recorded multi-axis motion signals during movement execution. IMU data were sampled at 4.65 Hz using the device's default configuration, without manual adjustment of the measurement range. The golden sample, obtained from the rehabilitation physician featured in the Health Buddy App exercise videos and who leads the Cancer Prehabilitation service, served as the reference motion pattern against which all measured signals were compared.

#### 2.3.2 Stage 2: scrolling wave method

**Figure 3:**
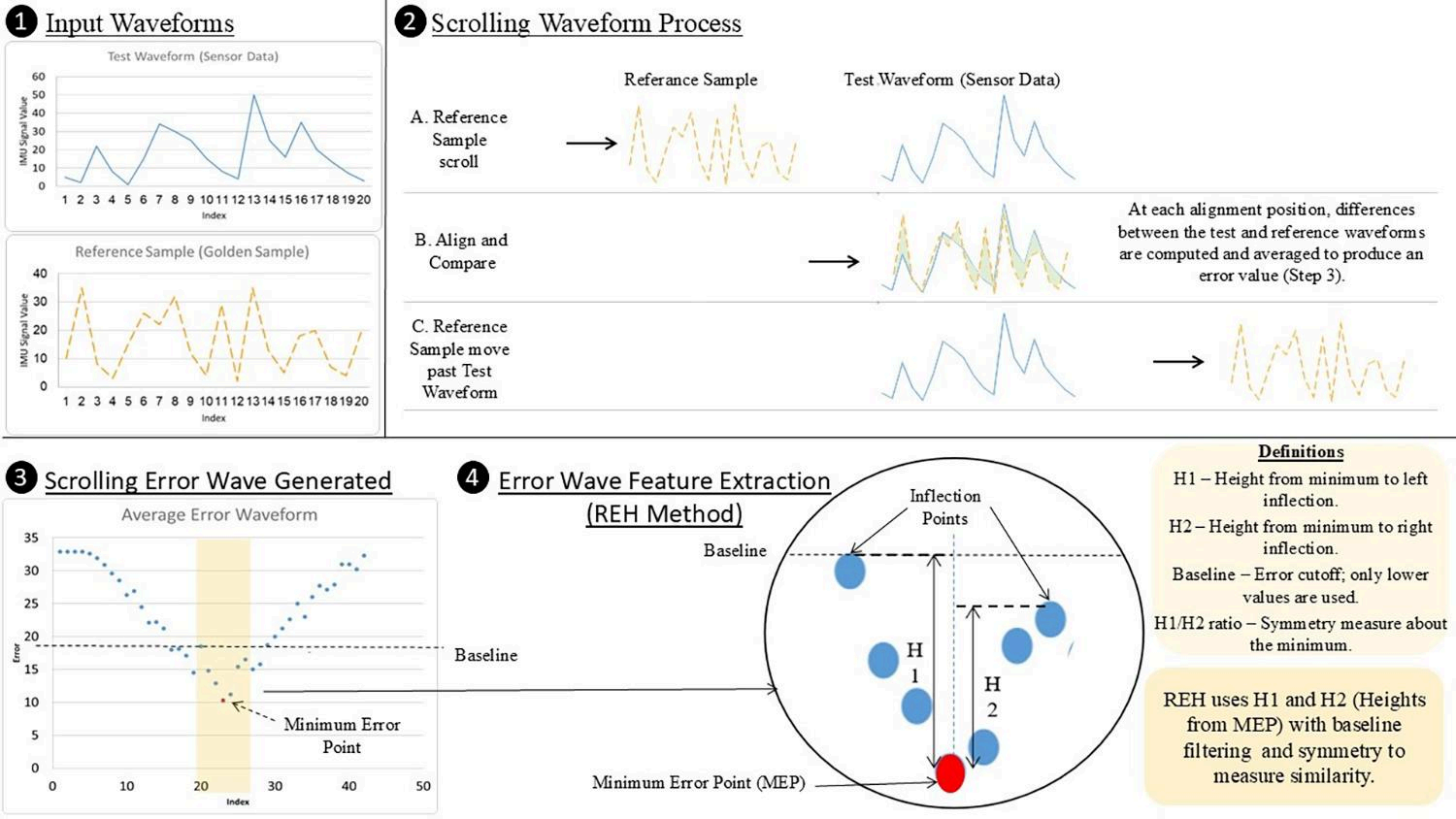
Scrolling wave method and REH scoring pipeline showing input waveforms, scrolling alignment, generation of the scrolling error wave (SEW), and feature extraction (H1, H2, and baseline) around the minimum error point (MEP).

The Scrolling Wave Method is a signal comparison technique designed to quantify the similarity between a measured sensor signal and a predefined reference signal. The reference signal was systematically shifted across the measured IMU signal to generate a Scrolling Error Wave (SEW), as illustrated in Figure 3 (Step 2). Error differences were plotted graphically and validated against recorded videos by investigators SST and ABZA.

#### 2.3.3 Stage 3: repetition error height (REH) scoring method

The novel Repetition Error Height (REH) scoring method, illustrated in Figure 3 (Step 4), operates directly on the SEW. Each repetition on the error graph was evaluated against four validation criteria:

1. H1 height threshold, defined as the vertical distance from the minimum point to the left inflection point.
2. H2 height threshold, defined as the vertical distance from the minimum point to the right inflection point.
3. a base threshold serving as a cutoff level that filters high-error regions, whereby values exceeding the baseline are excluded and values below them are used to assess alignment; and
4. the H1/H2 ratio, representing geometric symmetry around the minimum point.

Only repetitions satisfying all four criteria were considered valid. This approach enables complex motion waveforms to be decomposed and characterised using four parameters, rendering the method computationally efficient and suitable for real-time or embedded implementation. The process was subsequently coded and automated.

#### 2.3.4 Stage 4: automatic scaling and robustness

To accommodate natural inter-individual variability in movement execution — including motions of greater or lesser magnitude relative to the golden sample — the algorithm incorporates automatic scaling of the accelerometer and gyroscope signals along the *Y*-axis.

### 2.4 Algorithm validation

Twenty healthy volunteers were recruited for prototype validation. Each participant performed 7 exercises using the watch and tablet in the research unit under observation by SST and ABZA. Volunteers received exercise video demonstrations and provided return demonstrations before commencing. During the 30-repetitions session, participants were not interrupted even if form deviated from video instructions. Exercise form and repetition counts were visually observed and recorded.

Validation utilised on-site collected waveforms. Each volunteer completed 4 additional days of home exercises with provided exercise logs. Satisfaction surveys were completed upon device return.

### 2.5 Statistical analysis

The number of visually counted and algorithm-detected repetitions was reported as mean ± standard deviation (SD) and median with interquartile range (IQR). The algorithm detection rate was defined as the percentage of total visually counted repetitions that were detected by the algorithm for a given exercise type.

The compliance rate per exercise per day from day 2–5 was defined as (algorithm-detected repetitions/prescribed repetitions) × 100%, with a cap at 100% so overcounting does not inflate compliance. We reported the median and interquartile range (IQR) across all exercises per day from day 2–5 using the self-reported patient-logged number of repetitions or the objective algorithm-detected number of repetitions. Compliance heatmaps were used to visualise the median prehabilitation exercise compliance by type of exercise and day, based on participant log or algorithm.

To assess smartwatch usability, we adapted the validated patient version of the standalone mHealth App Usability Questionnaire (16) [MAUQ], by replacing ‘app’ with ‘watch’ in each of the 18 items. No other modification was made. The MAUQ was selected as the measured constructs apply directly to wearable interfaces and its translation to Chinese (17) and Malay (18) allows for wider assessment of the final prototype in a multi-ethnic population. Each item was on a 7-point Likert scale, with ‘1’ indicating strongly disagree and ‘7’ indicating ’strongly agree’. The mean scores and corresponding standard deviations for overall usability (items 1–18), ease of use (items 1–5), user interface and satisfaction (items 6–12), and system usefulness (items 13–18) were reported, with higher scores indicating higher perceived usability. A score of 5.0–5.9 was considered good usability and ≥ 6.0 was considered excellent usability. Scores for a participant were calculated only when ≥ 80% of items in the respective scale were available (overall ≥ 15/18; subscales: ≥ 4/5, ≥ 6/7, ≥ 5/6). All analyses were performed using R version 4.4.3 (R Core Team, 2024; R Foundation for Statistical Computing, Vienna, Austria).

## 3 Results

### 3.1 Participant characteristics

Twenty healthy volunteers aged 21–60 years with no known chronic medical conditions participated. The cohort comprised 13 females (65%) and 7 males (35%), representing Singapore's multi-ethnic population diversity.

### 3.2 Algorithm performance

Correlation between visual and algorithmic repetition counting ranged from 88.9% to 98.8% across all exercises (Table 1). The highest correlation was observed for squat chop (98.8%) and dig and flex (98.9%), whilst the lowest was for sit-to-stand (88.9%). Validation issues occurred only with participants Volunteer 1 and Volunteer 8, whose repetition counts exceeded ± 3 from visual observations.

**Table 1:** Visually observed and algorithm-detected repetition counts and detection rates across prehabilitation exercises.

Exercise type	Visually counted repetitions		Algorithm-detected repetitions		Algorithm detection rate (%)
	Mean ± SD	Median (IQR)	Mean ± SD	Median (IQR)	
Squat chop	30.0 ± 0.1	30 (30–30)	29.7 ± 1.6	30 (30–30)	98.8
Ice skater	30.0 ± 0.6	30 (30–30)	29.4 ± 2.4	30 (30–30)	97.9
Dig and Flex	30.1 ± 0.3	30 (30–30)	29.8 ± 0.4	30 (29.5–30)	98.9
March and Twist	29.9 ± 0.7	30 (30–30)	29.1 ± 1.7	29.5 (28.5–30)	97.3
Tap and punch	30.3 ± 0.5	30 (30–30.5)	27.3 ± 5.6	29 (26.5–30)	90.0
Sit to stand	30.1 ± 0.2	30 (30–30)	26.7 ± 6.8	29 (28–29.5)	88.9
Cross Punch	30.1 ± 0.2	30 (30–30)	28.4 ± 2.6	30 (28–30)	94.4

### 3.3 Case analysis: participant volunteer 1 (sit-to-stand exercise)

**Figure 4:**
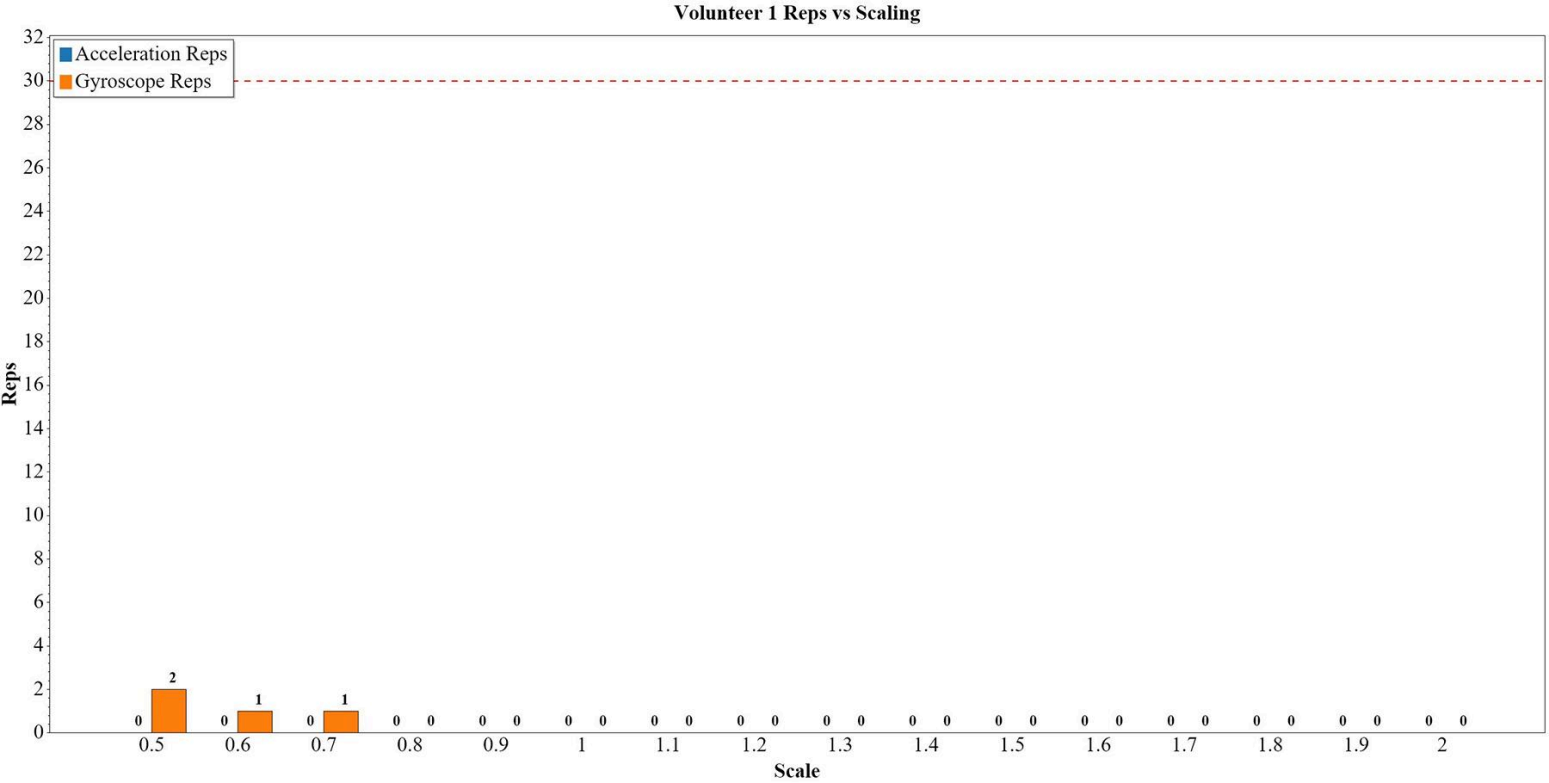
Scaled results (sit-to-stand volunteer1).

Volunteer 1 performed 30 sit-to-stand repetitions following video instructions closely and uniformly, yet only 2 repetitions were algorithmically detected. Both AccZ and GyroY signals demonstrated clear repeated motion evidence; however, waveform structure differed significantly from the golden sample. Scaling failed to resolve baseline offsets or shape differences, resulting in widespread REH feature validation failure (Figure 4).

**Figure 5:**
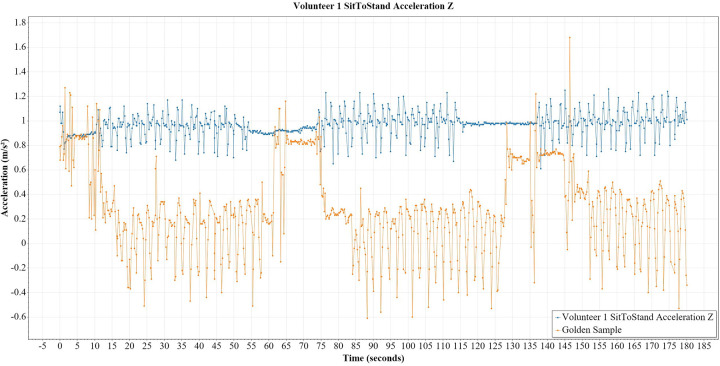
Accelerometer sit-to-stand volunteer 1 (blue-volunteer 1, orange-golden sample).

**Figure 6:**
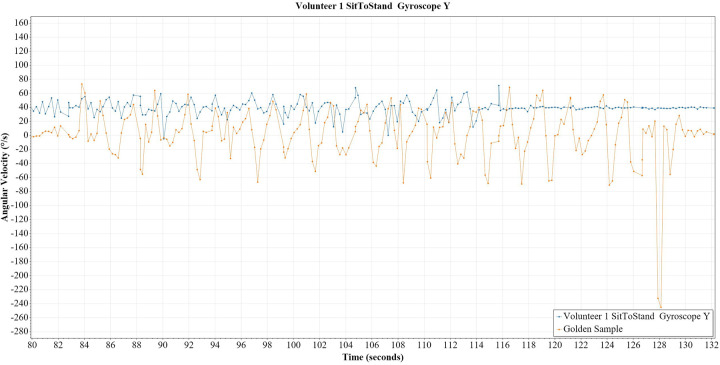
Gyroscope sit-to-stand volunteer 1 (blue-volunteer 1, orange-golden sample).

Comparative analysis revealed that whilst volunteer 1 followed exercise videos accurately, HCW1 had not performed exercises smoothly during golden sample recording, instead jerking upward quickly when standing, creating greater waveform amplitudes. Well-conditioned gluteus and quadriceps muscles enable slow, controlled movements, whereas momentum-dependent standing produces faster acceleration with greater H1/H2 asymmetry (Figure 5) and relatively small rotational movements (Figure 6).

### 3.4 Case analysis: participant volunteer 8 (multiple exercises)

Volunteer 8 demonstrated low repetition counts across multiple exercises (ice-skater, tap and punch, cross punch). REH waveform visualisation revealed significantly lower amplitudes compared to the golden sample. Volunteer 8 performed exercises in a gentle, consistent manner characteristic of her cultural background, with small-magnitude movements.

**Figure 7:**
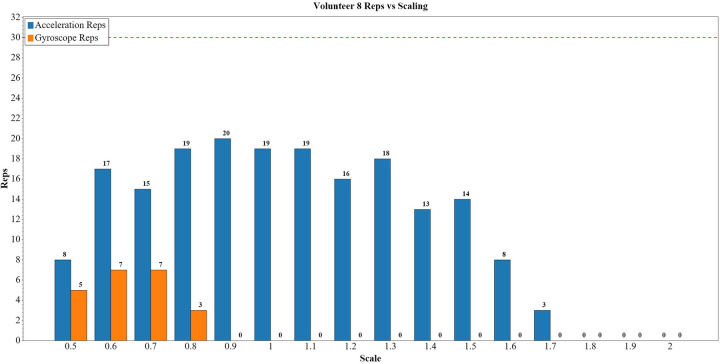
Scaled results (ice-skater volunteer 8).

**Figure 8:**
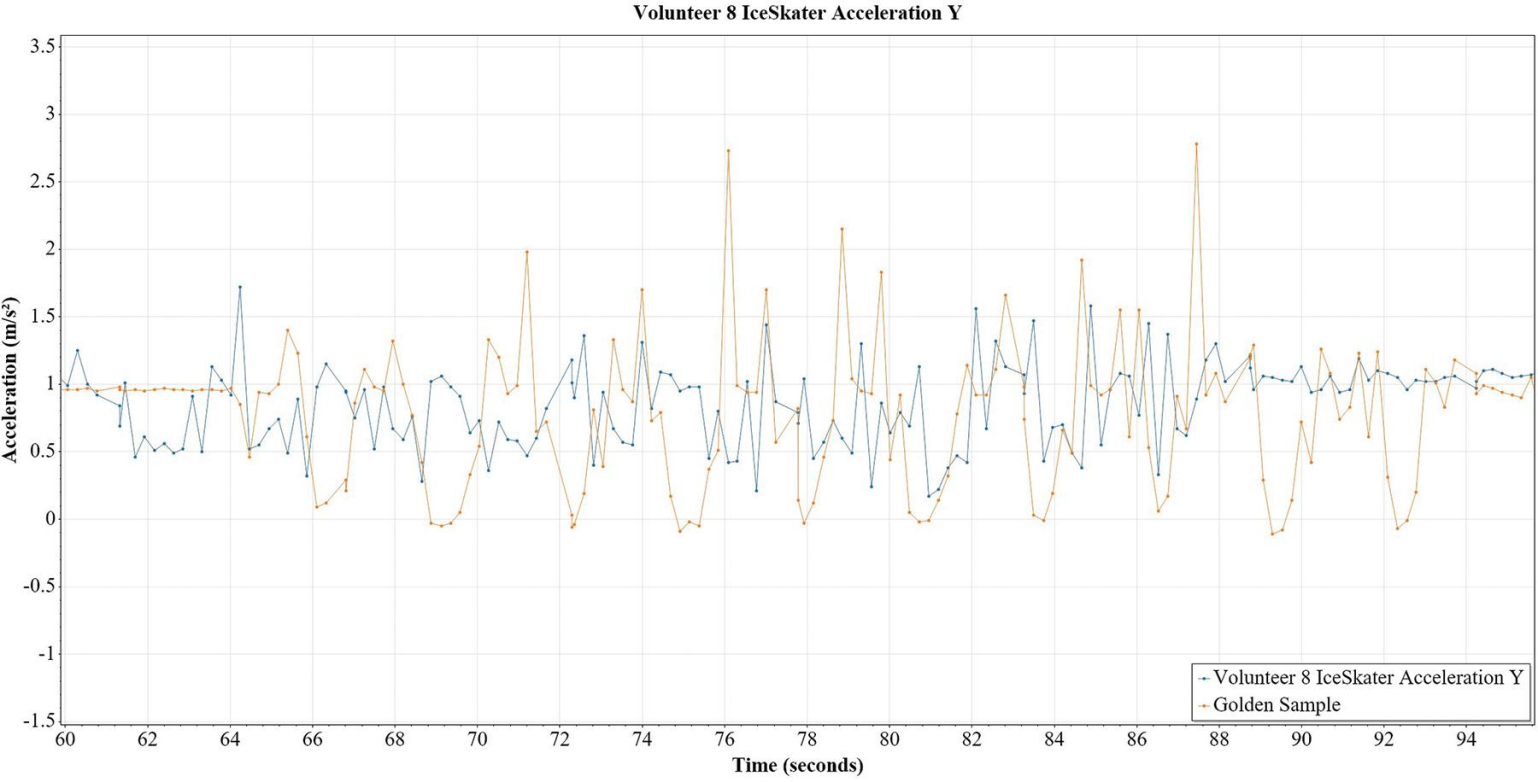
Accelerometer ice-skater volunteer 8 (blue - volunteer 8, orange - golden sample).

**Figure 9:**
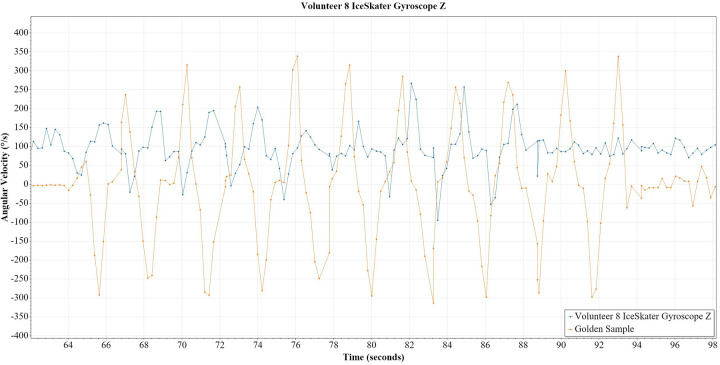
Gyroscope ice-skater volunteer 8 (blue - volunteer, orange - golden sample).

For the ice-skater exercise, Volunteer 8 showed significant repetition detection undercount with scaling providing minimal improvement (19→20 repetitions), representing a 33% shortfall relative to expected 30 repetitions (Figure 7). The golden sample exhibited clear, consistent cyclic patterns with pronounced peak-to-base separation, whilst Volunteer 8's waveform showed reduced and inconsistent amplitude with less uniform base-to-peak contrast (Figures 8, 9).

### 3.5 Home exercise compliance on day 2–5

**Figure 10:**
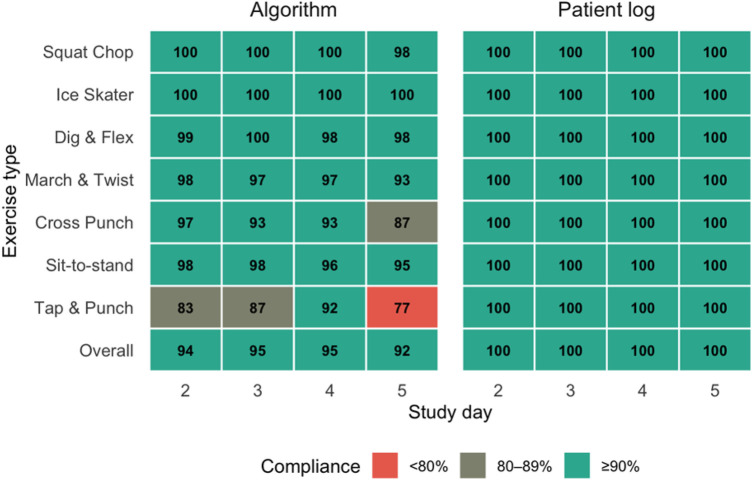
Heatmap of prehabilitation exercise compliance by type of exercise and study day. The left subplot shows percentage compliance based on objective algorithm detection while the right subplot shows percentage compliance based on subjective volunteer log. The compliance rate per exercise per day is defined as $\frac{Number\;of\;repetitions\;performed}{Number\;of\;repetitions\;prescribed\;(30)}*100\text{\%}$, with a cap at 100% so overcounting does not inflate compliance. Percentage compliance in each cell represents the median compliance across all 20 participants for a given exercise type on a given day.

Figure 10 shows the median prehabilitation exercise compliance by type of exercise and study day, based on objective algorithm detection or subjective patient log. The median overall compliance rates based on algorithm detection were 93.8% (IQR 90.1–97.6; day 2), 94.6% (89.4–96.7; day 3), 94.8% (88.3–96.4; day 4), and 92.1% (IQR 70.6–96.2; day 5). A lower median compliance rate on day 5 compared to day 2–4 was observed. Using patient log, the median overall compliance rates were 100% (IQR 100–100) from day 2–5. There were no dropouts during the home sessions.

### 3.6 Smartwatch usability

Table 2 shows the smartwatch usability scores using the adapted MAUQ. Seventy-five percent (15/20) of participants had available responses for ≥ 80% of items in the adapted MAUQ (ease of use subscale: 85% (17/20); interface and satisfaction: 80% (16/20); usefulness: 75% (14/20)). Among these participants, the mean overall score was 5.18 ± 1.03, corresponding to 60% (9/15) with a score of ≥ 5.0. The mean ease of use, interface and satisfaction, and usefulness scores were 5.99 ± 1.00, 5.14 ± 1.23, and 4.47 ± 1.54 respectively. When including all 20 participants in a sensitivity analysis, we observed similar mean scores (overall: 5.23 ± 1.07; ease of use: 6.09 ± 0.97; interface and satisfaction: 5.10 ± 1.26; usefulness [*n* = 19]: 4.49 ± 1.39).

**Table 2:** Smartwatch usability assessed using the adapted 18-item patient version of the standalone mHealth app usability questionnaire (MAUQ).

Subgroup and subscale	Mean ± SD	No. (%) of participants with score of ≥ 5.0
All participants		
Overall (*n* = 15)	5.18 ± 1.03	9/15 (60)
Ease of use (*n* = 17)	5.99 ± 1.00	15/17 (88.2)
Interface and satisfaction (*n* = 16)	5.14 ± 1.23	10/16 (62.5)
Usefulness (*n* = 14)	4.47 ± 1.54	5/14 (35.7)

The ‘overall’, ‘ease of use’, ‘interface and satisfaction’, and ‘usefulness’ scores are the mean scores of items 1–18, 1–5, 6–12, and 13–18 in the MAUQ, respectively. Each item is on a 7-point Likert scale, with ‘1’ indicating strongly disagree and ‘7’ indicating ’strongly agree’. Higher scores indicate higher perceived usability. A score of 5.0–5.9 is considered good usability and ≥ 6.0 is considered excellent usability. Scores for a participant were calculated only when ≥ 80% of items in the respective scale were available (overall ≥ 15/18 items; subscales: ≥ 4/5, ≥ 6/7, ≥ 5/6).

Of the 20 participants, six provided additional free-text comments about the smartwatch. A common theme was observed among four participants, who described a lack of direct feedback and visible information on the exercises. These included little to no on-watch information about exercise progress or completion confirmation, performance feedback including motion data visibility, and whether exercises were indeed logged. These four participants had mean (across items 13–18) usefulness scores of 1.3, 3.5, and 3.7 twice.

## 4 Discussion

### 4.1 Principal findings

This study demonstrates the feasibility of developing a customised wearable device for monitoring specific prehabilitation exercises. The prototype achieved high correlation (88.9%–98.8%) between visual and algorithmic repetition counting, indicating potential for objective exercise monitoring in home-based cancer prehabilitation programmes.

The algorithm's sensitivity in differentiating exercise forms represents both strength and limitation. Whilst it successfully identified exercises performed similarly to the golden sample, it demonstrated reduced accuracy for participants with different movement patterns, whether due to cultural influences, fitness levels, or technique variations. We recognize that the biomechanics, fatigue levels, and compliance patterns of a healthy volunteer are very different from a newly diagnosed older cancer patient awaiting surgery. However, once validated that the algorithm works, golden samples of various ages and fitness levels can be collected for future works.

Participant compliance, measured as the median percentage of algorithm-recorded repetitions relative to prescribed repetitions, exceeded 90% overall. However, compliance fell below 90% for specific exercises: tap and punch exercises on Days 2, 3, and 5, and cross punch exercises on Day 5. These deviations may reflect suboptimal exercise form during performance. Individual exercise analysis could identify form-related issues, though automated detection of such problems has not yet been implemented in the current programming. Participants informally reported that the home environment presented distractions that affected their adherence to prescribed timing, repetition counts, and proper form. Despite these limitations, this technology-based approach offers advantages over conventional prehabilitation methods, where patients may demonstrate complete inability to recall exercise techniques during pre-operative visits.

Usability assessment using the MAUQ revealed mean overall scores exceeding 5 for both ease of use and interface design, with a higher proportion of participants rating ease of use favourably ( > 5) compared to interface design (Table 2). However, only 5 of 14 participants (35.7%) rated the device's usefulness above 5. The utilisation of this affordable, versatile off-the-shelf device proved effective for algorithm development and prototyping purposes, with its ease of use demonstrating particular merit. Future interface refinements during the commercialisation phase will likely be necessary to achieve broader user acceptance. The limited perceived usefulness may be attributed to the absence of real-time feedback mechanisms, such as cloud connectivity or on-screen score display to participants. The current project scope focuses on prototype robustness testing, with feedback loop implementation planned for subsequent phases requiring additional resources. These usability findings provide valuable insights for addressing user experience considerations in future development iterations.

### 4.2 Comparison with prior work

Current commercial wearables focus primarily on aerobic exercise detection and step counting (15), with limited capability for strength training exercise recognition. Our prototype addresses this gap by specifically targeting dynamic, compound, and plyometric exercises essential for cancer prehabilitation. Unlike existing devices, our system provides exercise-specific monitoring with technique assessment capabilities.

Literature review identified Exersense (19) as the most comparable wearable device, capable of measuring five exercises: running, walking, jumping, push-ups, and sit-ups through segmented measurement approaches. However, these exercises are not typically prescribed for sedentary elderly populations and do not align with standard cancer prehabilitation protocols.

Additional studies have employed vision-based exercise recognition systems, primarily for sports applications including tennis and baseball (20, 21), which typically require three-dimensional analysis. Vision-based approaches are constrained by their requirement for dedicated locations and specialised equipment setup, limiting their practical applicability.

Previous work by Morris et al. (22) developed the RecoFit system for movement-repetition-based exercise recognition, utilising an arm-worn inertial sensor to monitor gymnasium-based exercises including crunches, rowing machine exercises, kettlebell swings, shoulder presses, and Russian twists. However, gymnasium-based training protocols may present barriers to participation among sedentary elderly populations and are therefore unsuitable for cancer prehabilitation programmes. Additionally, the counting methodology employed appears computationally complex.

In contrast, the wave analysis approach implemented in this smartwatch algorithm offers computational simplicity and resource efficiency, requiring minimal processing power and memory allocation whilst maintaining effectiveness for the target exercise types.

### 4.3 Limitations

Several limitations warrant consideration. The golden sample was derived from a single individual whose technique may not represent optimal form for all participants. The algorithm requires refinement to accommodate cultural and individual movement as well as exercise speed variations without compromising accuracy. The small sample size limits generalisability, and longer-term compliance data are needed to assess real-world applicability. Additionally, the study was conducted in a controlled environment, and real-world performance may differ, as the validation of the algorithm was conducted on healthy volunteers and not cancer patients. However, the doctor featured in the video and who was also the golden sample had reduced his intensity of exercise to suit the patients that he is seeing in his service.

### 4.4 Future development

The prototype has the potential to address a critical need in cancer prehabilitation by providing objective exercise monitoring capabilities. This technology could enhance programme effectiveness by enabling healthcare providers to monitor patient compliance remotely and provide targeted feedback. The system's ability to differentiate exercise quality could support personalised intervention strategies.

Future development priorities should encompass several key areas to enhance the clinical utility and generalisability of this monitoring system. Algorithm refinement represents the primary focus, with emphasis on accommodating diverse movement patterns whilst preserving detection accuracy. Machine learning approaches, though not without its challenges (23–25), offer promising solutions through the incorporation of multiple reference standards that represent varied demographic groups and cultural movement styles. Expanding the training dataset to include larger, more diverse population samples would strengthen algorithm robustness and reduce bias towards specific movement patterns.

The development of population-specific golden samples, stratified by demographic characteristics such as age, ethnicity, and fitness level, presents an alternative approach to address inter-individual variability in exercise execution.

In addition, the correlation of specific movements in the exercises to sensor measurements can be used to model an exercise objectively and may be more accurate than golden samples. This approach provides clearer insights into the participants’ movements and reveal deviations in performing the exercises and automatically suggests corrections. Furthermore, new exercises can be modelled in a shorter time after verification with healthcare workers.

Additionally, seamless integration with existing healthcare information systems will be crucial for clinical implementation and workflow adoption (26, 27). Validation studies in actual cancer patient populations, rather than healthy volunteers, represent an essential next step to establish clinical efficacy and safety. These studies should evaluate not only technical performance but also patient acceptance (6, 28), adherence rates, and clinical outcomes in the target population. Furthermore, longitudinal studies examining the relationship between objectively measured exercise compliance and clinical endpoints such as functional capacity, treatment tolerance, and recovery outcomes would provide valuable evidence for the system's clinical value proposition (7).

## 5 Conclusions

We successfully developed a prototype smartwatch capable of monitoring dynamic, compound, and plyometric exercises with high accuracy for participants whose movement patterns align with the reference standard. Whilst the algorithm demonstrates high sensitivity for exercise form differentiation, further refinement is needed to accommodate diverse movement patterns and cultural variations in exercise execution.

## Data Availability

The raw data supporting the conclusions of this article will be made available by the authors, without undue reservation.
